# Local perceptions of foodscapes and their representation in visual and texts: A visual content analysis of photography in Japanese coastal area

**DOI:** 10.1016/j.crfs.2025.101197

**Published:** 2025-09-12

**Authors:** Masaki Uchida, Rei Sugawara, Ryo Kohsaka

**Affiliations:** a Graduate School of Agricultural and Life Science, The University of Tokyo, 1-1-1 Yayoi, Bunkyo-ku, Tokyo, 113-8657, Japan; b Faculty of Business Administration, Ishinomaki Senshu University, 1 Minamizakaishinmito, Ishinomaki, Miyagi, 986-8580, Japan

**Keywords:** Foodscape, Food, Landscape, Local environments, Visual representations, Textual representations

## Abstract

The term “foodscape” has recently gained prominence in academic discourse, fostering interdisciplinary research into the relationship between food and local environments. This study focuses on the material aspect of foodscapes and aims to clarify how local residents perceive them. We also analyze differences between visual and textual expressions. Targeting university students in the coastal area of Japan, Ishinomaki district, Miyagi Prefecture, the research employs visual materials (photographs) and textual responses (questionnaire).

First, focusing on landscape aspects of foodscape—particularly visual views—we conducted photo-based analysis based on expert judgement using visual parameters: visual object, viewpoint, visual distance, and perspective. Participants photographed scenes associated with “foodscapes.” Multiple Correspondence Analysis (MCA) categorized images into five types. Results showed relationships between food categories and photo composition, but seafood—an important local industry—appeared across almost all types regardless of visual parameters.

Second, comparative analysis of photographs and textual responses elucidated how food and place are jointly imagined in local foodscapes. Compared with a preliminary survey of regional specialties and landmarks, photographs showed decreased seafood imagery and increased restaurant and retail scenes, mostly in urban areas. Findings suggest that when foodscapes are visually expressed through non-verbal media such as photography, imagery emphasizes familiar daily settings—particularly sites related to food production and distribution—rather than iconic regional foods or tourist landmarks.

The analytical perspective of this study provides a framework for developing context-sensitive approaches reflecting residents’ sense of place in food tourism, regional planning, and cultural landscape research.

## Highlights

• This study examined foodscapes using both visual and textual data.
• Seafood, a specialty food in coastal area, was depicted in diverse photographic compositions.
• Foods with low local relevance were mainly photographed on its own.
• Photographs, as non-verbal representations, emphasized daily foodscapes.

## Graphical abstract

**Figure:**
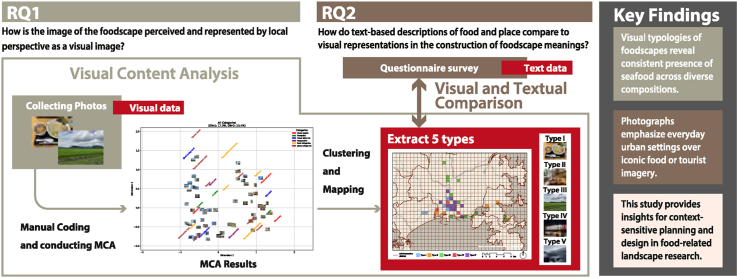

## 1 Introduction

### 1.1 Foodscape, food landscape, and food and landscape: the focus of this study

The “foodscape” concept has, since first being introduced into academic literature by Yasmeen (1995) in 1995, been employed as a framework for discussing the socio-spatial inequalities inherent in food, local environments, public health, and food systems (Vonthron et al., 2020). Although its definitions and applications vary, the term is etymologically derived from a combination of “food” and “landscape.” Adema (2010) defines foodscape as “a marriage between food and landscape, both the conceptual notion (idea) of landscape and actual, physical landscapes.” In this sense, foodscape is not merely a physical space, but a spatially recognized phenomenon shaped by people's gaze and perception of food.

What is the nuance of different terminologies surrounding foodscapes and similar terminologies? Within the broader foodscapes discourse, “food landscape (Sobal and Wansink, 2007; Kawai, 2020)” is focused more on particular foods and the tangible spaces surrounding them—such as shops, markets, ritual sites, farms, coastlines, ethnic enclaves, and urban areas. In a related formulation, Sobal et al. (2007) (Sobal and Wansink, 2007) defined the food landscape as “macro-scale food environments,” stating that “the food landscape considers the foods within the sum of all elements in larger landscapes, and food landscapes shape the foods that enter a household and which can later be eaten by particular individuals (Sobal and Wansink, 2007).” These spaces are materially grounded in food's production, distribution, and consumption. They may also be imbued with profit-oriented intentions or political ideologies, rendering them physical environments layered with affective and symbolic meanings.

The term “food and landscape” was used as the title of an article (Roe et al., 2016) and editorial (Roe, 2016), representing an approach grounded in the spatial perspective of landscape and urban planning. The findings of Roe et al. (2016) (Roe et al., 2016) in the Journal Landscape Research can also be interpreted as foundational data for diagnosing how consumers interact with specific “food landscapes”.

Historically, foodscape first gained traction in the visual arts, particularly in painting and photography (Kawai, 2020; Roe et al., 2016; Mikkelsen, 2011). Its etymological roots and disciplinary trajectory thus underline its close association with visual perceptions. Therefore, the relationship with vision can be regarded as one of the central elements of the foodscape concept. Since Yasmeen's pioneering work in 1995 (Yasmeen, 1995), the concept has expanded across a wide range of disciplines, including geography, sociology, anthropology, architecture, and public nutrition in scientific literature. In practice, the usage of coined words in form of suffix “-scape” has been in increase extended. These terms are frequently used metaphorically as a “gaze on food” rather than referring to visual properties *per se*.

Research employing the notion of “foodscape” can be broadly classified into four major approaches: spatial approaches, social and cultural approaches, behavioral approaches, and systemic approaches (Vonthron et al., 2020). For example, spatial approaches frequently apply statistics and GIS (Geographic Information Systems) to analyze food accessibility and its implications for health (e.g. (Cummins and Macintyre, 2002; Tharrey et al., 2024);). Social and cultural approaches discuss foodscapes as outcomes of history, culture, and social justice, exemplified by Yasmeen's geographical studies of Bangkok (Yasmeen, 1995, 1996) or research examining the interrelations of people, food, and place with a focus on communities (e.g. (Holloway and Kneafsey, 2000; Miewald and McCann, 2014),). Systemic approaches, in turn, address global debates on food sustainability and security, such as analyses of regional food policies aimed at improving the sustainability of foodscapes through the re-localization of food chains (Morgan and Sonnino, 2010). In recent works, discourse analyses of public opinion on food safety using big data have also been conducted, propelled by advances in machine learning (Zhang et al., 2023).

Behavioral approaches focus on the sensory perceptions of recipients. For instance, studies have examined children's recognition of foodscapes through the construction of cognitive maps (Earl, 2018), or analyzed how visual, olfactory, and tactile characteristics of retail stores influence purchasing behavior and consumer choices. By doing so, behavioral approaches analyze the perception of foodscapes by individuals through the visualization of their behaviors. We seek to offer insights by integrating behavioral and spatial approaches—interpreting community-based perceptions through their spatial configurations in this paper moving beyond the fourfold typology outlined above. To link spatial and behavioral aspects, we analyze both shooting locations of photographs and public perceptions. The perspective adopted in this paper, we closely aligns with the scholarly viewpoint articulated in the notion of “food and landscape” as employed by Roe et al. (Roe, 2016).

### 1.2 Foodscapes as the intersection of food, tourism, and landscape

Foodscape discourses are applied in connection with tourism and branding, with a focus on visitors, attraction, and management, as well as on visual appearance and experiential dimensions. Studies in the area conceptualized foodscapes as stages for tourist experiences (Björk and Kauppinen-Räisänen, 2019), identified diverse forms such as globalized, staged, or authentic encounters (Zhu et al., 2022), and emphasized their dynamic co-creation with local communities (Park and Widyanta, 2022). In parallel, reviews of sustainable tourism highlight that communities and local residents are increasingly recognized as crucial stakeholders, since they directly experience the challenges of environmental degradation and changing living conditions (Agarwal et al., 2024; Li et al., 2019). Communities and local residents develop relationships with their environment. In addition, an increasing number of studies have employed visual materials to explore the nexus between tourism and foodscapes (Goodman and Jaworska, 2020; Lee et al., 2025; Tang et al., 2024; Xiao et al., 2022). However, because such analyses frequently rely on promotional food photography, tourist-generated phtographs, or visually appealing dishes designed for social media, In other words, existing studies in food tourism study tended to focus more on visitors’ perspectives.

Yet, such photography may not necessarily reflect the foods with which local residents maintain strong attachments. Thus we analyze local perceptions as the primary focus, in order to provide basis insights to capture community-rooted foodscapes that reflect residents’ identities within the broader framework of sustainable tourism, thereby contributing to the advancement of sustainable food tourism.

Urban and landscape research has increasingly focused on the relationship between food culture and the landscape of cities and rural mountain areas. These studies aim to visualize food's role in shaping the form, function, and identity of place (Roe et al., 2016; Fontefrancesco et al., 2023). Other strands of research have examined urban food growing and food self-sufficiency, seeking to combine these themes with urban design and landscape planning (Coles and Costa, 2018; Sylla et al., 2022). However, much of this work tends toward typological or descriptive analysis aimed at contributing to landscape characterization inventories, or technical issues such as those used in Landscape Character Assessment (LCA), thereby emphasizing objective features that define landscape and regional identities. Relatively little attention has been paid to analyzing foodscapes from the perspective of human perception and experience.

**Fig. 1:**
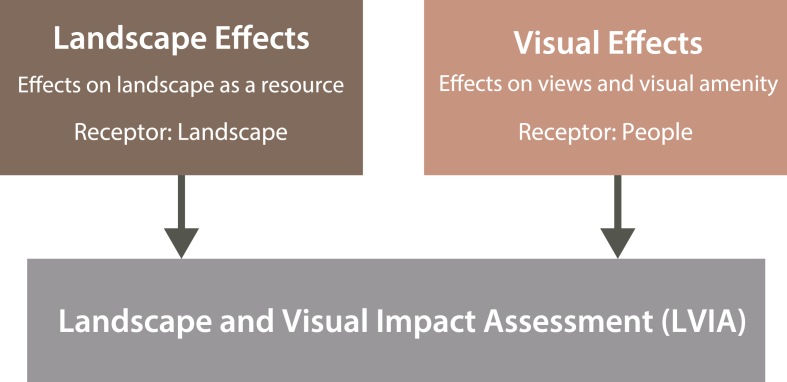
The framework of Landscape and Visual Impact Assessment (LVIA), source: Landscape Institute and Institute of Environmental Management and Assessment (2013).

Meanwhile, the European Landscape Convention (Council of Europe, 2000) defines landscape as “an area, as perceived by people, whose character is the result of the action and interaction of natural and/or human factors.” Similarly, the Guidelines for Landscape and Visual Impact Assessment (Landscape Institute and Institute of Environmental Management and Assessment, 2013), widely used in landscape assessment, emphasize the need to evaluate both landscape and visual impacts when considering the effects on a given landscape (Fig. 1). If we apply this perspective to foodscape's “landscape” component, a deeper understanding of foodscapes requires the subjective and experiential dimensions—how people perceive, remember, and assign meaning to these spaces in addition to objective elements. By incorporating these subjective aspects, it becomes possible to view social contexts and perceptions of foodscapes as landscapes from perspectives of locals—an area largely underexplored in existing foodscape literature.

Regarding analytical methods concerning visual aspects, the field of landscape studies has long employed approaches such as Visual Quality Assessment (VQA) and Visual Impact Assessment (VIA), which evaluate perceptual and psychological values (e.g. (Zube et al., 1982a),) through the use of various forms of visualizations (Gobster et al., 2019). These visualizations include simulation-based techniques such as photo montages, computer graphics, augmented reality, and virtual reality (Lange, 2011), as well as quantitative analyses that utilize numerical indicators such as viewing angles and viewsheds (Hurtado et al., 2004; Manchado et al., 2015). The last two decades have seen the revitalization of visual evaluations’ academic significance, driven by the rise of renewable energy projects including wind-farm-related facilities and rapid advancements in image processing technologies (Gobster et al., 2019; Miyawaki and Uchida, 2023).

Furthermore, approaches incorporating public participation—e.g., those analyzing communication among participants regarding forest landscapes using statistical methods—have been developed in other fields of environmental studies (Kohsaka and Flitner, 2004; Kohsaka and Handoh, 2006). Additional methods—including Visual Content Analysis (VCA)—that analyze the content of visual media such as magazines, advertisements, and social networking services both quantitatively and qualitatively, have also evolved (Araujo et al., 2020; Bell et al., 2001; Rose, 2001). Alternatively, studies centered on visual imagery involving the dual semantic layers of “food” and “landscape” remain extremely limited. Accordingly, established analytical frameworks able to address these layers’ interconnection is lacking.

VIA tends to focus on tools and techniques that enhance accuracy and precision for estimating visual impacts' magnitude. Contrastingly, VQA studies emphasize methodological issues such as scales’ reliability, consensus among stakeholder groups, and the selection of indicators and model specifications for theoretical development and prediction (Gobster et al., 2019). Given these limitations in scope, it is necessary to adopt a more participatory and public approach to unfold local perceptions—which exist on a different plane from the impacts of change or aesthetic quality (Zube et al., 1982b).

This study builds on the perspective developed in the rich literature related to VQA and VIA primarily cultivated within landscape studies and aims to apply the approach to foodscapes. We seek to clarify the gap of foodscapes perceived by the tourists or visitors and those experienced by the local residents. We analyze the associated meanings of foodscapes as lived by the local residents. By combining different methodologies, we analyze the perceptions and discourse of foodscapes, rather than focusing solely on image data in order to provide methodological insights to foodscape studies.

### 1.3 Objective

Building on the discussions outlined above regarding the conceptualization of foodscapes and their intersections with tourism and landscape studies, this study seeks to address the underexplored area of community-centered perceptions of foodscapes. While prior research has largely focused on visual representations geared toward tourists or typological descriptions of landscapes, relatively little attention has been paid to understanding how local residents perceive, remember, and assign meaning to the food and landscape around them. By integrating visual and textual analyses, this study aims to highlight foodscapes as experienced and valued by local communities, without focusing on those staged for external audiences.

**Fig. 2:**
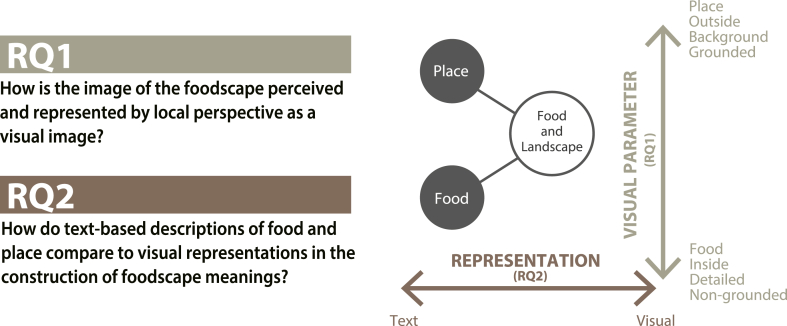
Diagram of research questions.

Therefore, as illustrated in Fig. 2, this study poses two research questions, and the corresponding objectives are as follows:

1. Examine the interactions of food and landscape by categorizing photographs taken by local residents using visual content analysis across multiple visual parameters. In this objective, we aim to apply methodologies from both tourism and landscape studies to foodscape research, facilitating theoretically grounded interpretations of spatial perceptions related to food, and elucidating patterns and spatial relationships specific to particular local contexts.
2. Clarify meaning-making in foodscapes by comparing textual representations of local specialties and iconic sites with the visual representations derived from the categorized photographs. In this objective, we seek to illuminate subjective and culturally embedded perceptions of food and place, capturing the multilayered meanings that residents attach to their local foodscapes, thereby advancing research on community-rooted foodscapes that are not staged or designed for external audiences but are genuinely embedded in local daily life and practices.

Through these objectives, the study contributes to advancing a perception-based understanding of foodscapes, offering insights that complement existing research focused on tourism, branding, and landscape typologies while emphasizing the authentic, resident-centered character of local foodscapes.

## 2 Material and method

### 2.1 Research subjects

#### 2.1.1 Research area

**Fig. 3:**
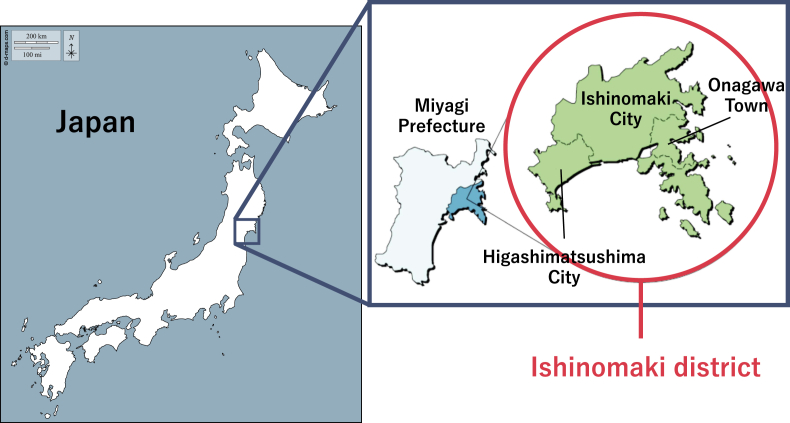
Ishinomaki district, source: Ishinomaki district integrated administration of a large region office work association.

The study area selected for this research is the Ishinomaki district (Fig. 3)—a region comprising an intermunicipal administrative entity within Miyagi Prefecture, Japan, encompassing three local governments: Ishinomaki City, Higashimatsushima City, and Onagawa Town. Portions or the entirety of each of these municipalities fall within the Ishinomaki Greater Urban Planning Area—a statutory planning unit governed by a shared development framework.

In Japan, urban planning areas are designated by prefectural governors not based on administrative boundaries, but according to the necessity for comprehensive development, improvement, or conservation of areas functioning as integrated urban units. Thus, these three municipalities’ inclusion within a single planning area reflects the substantial socio-spatial coherence among them.

This district suffered extensive damage in 2011's Great East Japan Earthquake. In the course of post-disaster recovery, the reconstruction and revitalization of local industries—particularly fisheries—along with the enhancement of tourism based on natural and historical assets such as Matsushima—a nationally designated Special Place of Scenic Beauty—have been central pillars of regional planning (Miyagi Prefecture, 2025).

Given this dual focus on the promotion of food-related industries and the conservation and utilization of scenic landscapes, the Ishinomaki district constitutes an appropriate case study site for examining the food–landscape interrelation.

#### 2.1.2 Research participants

This study's participants were 101 undergraduate students enrolled in either the Department of Business Administration or the Department of Information Management at Ishinomaki Senshu University, located in Ishinomaki City. This selection was made based on the consideration that, in Japan, food and landscape are often treated as distinct academic fields; therefore, involving individuals with specialized knowledge in only one of these domains might have introduced bias into the results. By selecting students without a specific disciplinary focus on either domain, a more balanced perspective could be obtained.

Furthermore, given Japanese university students’ high smartphone usage, they were deemed an appropriate demographic for photograph-based data collection. As the winter season is generally unsuitable for photographing landscapes due to snowfall and adverse weather conditions, the study was conducted during summer.

### 2.2 Visual content analysis

In the first phase, this study applied VCA—an analytical approach utilizing visual media such as photographs and videos, commonly applied in the social sciences. VCA encompasses both quantitative and qualitative approaches, and recently its application has become increasingly hybridized depending on the research objective (Li et al., 2023). Regarding image recognition—used as a quantitative approach—recent advancements in artificial intelligence (AI) have significantly advanced the field of computer vision (CV), which deals with automatic image recognition. This has led to the development and application of specialized models not only for image classification but also for object detection and segmentation across various fields (Kirillov et al., 2023; Wang et al., 2023). In the fields of tourism and place branding, more and more research has been employing image recognition models to analyze visual representations of food and place (Xiao et al., 2022; Li et al., 2023; Cerić et al., 2024; Chen et al., 2023; Cristina and Cristina, 2024). However, there are also some limitations in these methodological approaches found in the literature. While these machine learning techniques are well-suited to the analysis of large-scale datasets, they exhibit limitations when applied to context-specific and nuanced classification tasks. Particularly, when dealing with landscape, the use of numerical representations may risk implying misleading relationships, especially when the meaning is overly concretized (Landscape Institute and Institute of Environmental Management and Assessment, 2013).

Reflecting these, the present study employed a qualitative approach to accurately capture the perception of food-related landscapes embedded in a particular region. Specifically, we utilized photo elicitation methods and multiple correspondence analysis (MCA) to summarize the patterns of public perception regarding food and landscape. These findings are intended to serve as foundational data for future analyses employing machine learning techniques.

**Fig. 4:**
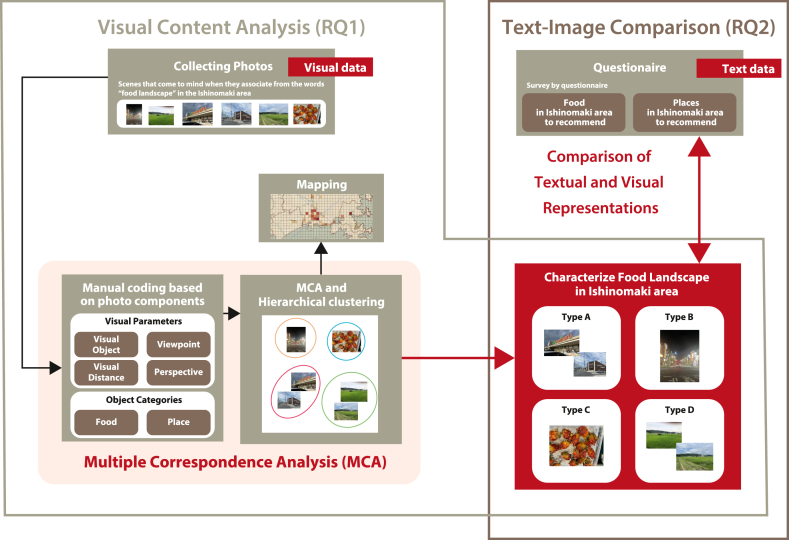
Research flow.

To address the research questions and corresponding objectives presented in Fig. 2, this study employed two primary methods with the above-mentioned participants: a visual content analysis using visual parameters, and a comparative analysis of visual and textual representations. The overall research process is illustrated in Fig. 4. The following sections detail each of these methodological approaches.

#### 2.2.1 Collecting photographs using the photo-projective method

The Photo-Projective Method (PPM) was originally proposed by psychiatrist Masaaki Noda, as a projective analytical technique using photographs to explore environmental worlds (Noda, 1988). In PPM, photographs are conceptualized as “tools for evaluating space through inner consciousness (Hisa and Narumi, 1992)”. While PPM broadly falls under the umbrella of VCA, a distinctive feature of this method is that researchers provide a thematic prompt, and participants freely select and capture visual images that come to mind in response. Consequently, the resulting photographs represent a free expression of internal representations and mental landscapes. This approach avoids the ambiguity often encountered in big data-driven photographic datasets with unclear shooting intentions, making PPM an effective means to capture subjective perspectives.

In this study, participants were asked to take approximately three photographs each, depicting “scenes that come to mind when thinking of the ‘foodscape’ of the Ishinomaki district.” This abstract prompt was designed to encourage participants to consider how they associate food with landscape and to express these associations visually. The photo-taking was conducted from July 16 to July 30, 2024. Participants were asked to use smartphones for photography, given the ease of acquiring geolocation data. The framing and viewpoints of the photos were left entirely to the participants' discretion. Although numerous prior studies and guidelines specify standards for framing (Kunii, 2019; Ota et al., 2023; Scottish Natural Heritage, 2017), this study focused on the subjective recognition of landscape and regarded the way the scene is framed as part of the visual representation itself. Therefore, consistent with the original PPM framework, the analysis was based on freely taken photographs. Along with the photographs, brief comments regarding the reasons for choosing the shots were also collected.

#### 2.2.2 Extracting photo components

To categorize the collected photographs according to the visual aspects of landscape in line with the objectives of this study, an analysis of the compositional elements within the photographs was first conducted. Visual elements of landscape include, for example, the “form, line, color, and texture” used in the Visual Contrast Rating by the United States Bureau of Land Management, as well as the categories “Landform, Landcover, Informational Variables, Perception-Based Variables” employed by Kaplan (Kaplan et al., 1989). The primary focus in this paper was on exploring public impressions rather than the quality of the landscape. Therefore, to capture only the visual composition, we referred to landscape comprehension models that can explain traditional Japanese borrowed scenery (*shakkei*), Pure Land-style gardens, and Hakkei-style landscape appreciation methods (Shinohara, 1982), as well as existing research (Kaplan et al., 1989; de la Fuente de Val et al., 2006; Zeng et al., 2022), and organized those models into visual parameters. Given the limited sample size, we hypothetically selected four visual parameters: visual object, viewpoint, viewing distance, and visibility. The criteria for judgment and categories of these visual parameters are shown in Table 1. Since distance information is not included in the photographs, viewing distance was treated based on the apparent size of visible elements, referring to Japanese literature (Shinohara, 1982).

**Table 1:** List of visual parameters and judgement criteria.

Visual Parameters		
variables	categories	judgement criteria
Visual object	foodonly	Due to sample size limitations, visual objects were categorized as food only, location only, or both. Although some objects, such as farmland, could be interpreted as food, they were classified as location due to their geographic situatedness.
	placeonly	
	food and place	
Viewpoint	inside	To account for differences between internal and external perspectives, and between scenes and sequences, viewpoints were classified as inside, outside, and vehicle categories.
	outside	
	vehicle	
Visual distance	detailed	In this research, visual distance refers to the relative distance to the main object. Following prior studies and guidelines, we classified images into detailed, fore_midground, and mid_background, based on the perceived size of the main object within the frame. Food-centric and very close-up images were categorized as detailed.
	fore-midground	
	mid-background	
Perspective	grounded	Perspective was coded separately from visual distance. Based on the figure-ground relationship, ground-like subjects were considered to have perspective, while figure-like ones were judged by the inclusion of broader scenery. Building on de la Fuente de Val et al. (2006), we expanded the definition of perspective to include non-landscape images (e.g., food, interiors), by assessing whether the viewpoint captures spatial context.
	non-grounded	

Alongside the visual parameters, the photographic intent was also considered by semantically categorizing both the depicted or associated food and place elements (Table 2). When food appeared in the photograph, the corresponding food category was assigned; similarly, when a place appeared, the corresponding place category was assigned. There were cases where either food or place was absent. In such instances, for photographs depicting only a place, the associated food was inferred, and for those depicting only food, the place of photography was identified based on the photograph and accompanying comments. These were then incorporated as variables.

**Table 2:** List of categories of visual objects.

Object Categories	
variables	categories
Food Categories	noodle
	seafood
	agricultural
	and forestry
	fastfood
	on-campus
	dining
	multiple

Place Categories	restaurant
	retail store
	street
	commercial hub
	campus
	green space
	open water/port area
	others

It is generally understood that humans can distinguish approximately seven different classes at one time (Miller, 1956; Rosch and Lloyd, 1978). Additionally, to ensure the subsequent multiple correspondence analysis’ reliability and reduce categories with an extremely small number of samples (five or fewer), the number of categories was aggregated and adjusted.

Regarding variables’ aggregation, the food category “agricultural and forestry” included not only farmland and crops, but also livestock products and mountains. The category “multiple” consolidated items attempting to depict various types of food, such as facilities like complexes handling diverse foods or kitchens. Although the “on-campus dining” category contained various types, it was separately categorized because the place aspect was often emphasized over food diversity as a representation of everyday food scenes.

For the place categories, farmland and mountains should ideally be categorized separately, although sample size constraints meant they had to be aggregated into a “green space” category. According to Japan's Urban Green Space Act, waterside areas are also considered green spaces, but given Ishinomaki's prominence in fisheries, aquatic products and water areas were semantically distinguished and assigned a separate category.

#### 2.2.3 Multiple correspondence analysis (MCA)

Based on the manual coding of visual parameters as well as food and place categories, we conducted MCA—an effective method for handling multiple categorical variables. It calculates category scores for each assigned category and arranges these categories as coordinates on a two-dimensional plane. Similarly, samples are also assigned coordinates in a multidimensional space, and, typically, the two axes with the highest contribution rates are used to plot the samples on a two-dimensional plane. In this study, the results of manual coding were analyzed using the MCA class from the prince Python library (version 0.7.1).

### 2.3 Comparison between visual and textual representations

The VCA-derived results were positioned as non-verbal representations, and a complementary language-based survey using questionnaires was also conducted as a comparative sample. The questionnaire employed in this study comprised the following items: (1) local specialty foods in the Ishinomaki district that respondents would recommend to friends or family visiting the region for the first time; (2) local places in the same region that respondents would recommend under the same conditions; and (3) whether respondents were familiar with the term “foodscape.” Items (1) and (2) were designed to clearly capture region-specific foods and places in Ishinomaki from the perspective of individual subjective evaluation. Notably, the photographs and questionnaire responses were not linked on an individual basis but were collected as separate datasets from the same population.

The obtained results were analyzed by comparing the proportion of each response relative to the entire dataset, and differences between verbal and non-verbal representations were discussed.

## 3 Results

### 3.1 Results of visual content analysis

**Fig. 5:**
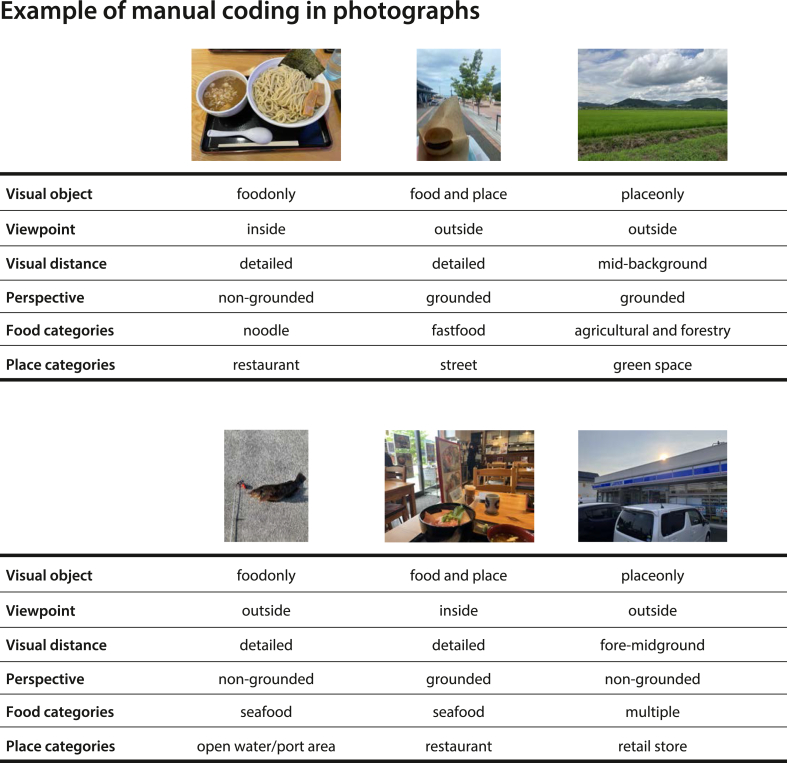
Example of manual coding in photographs.

A total of 115 photographs were obtained by filtering the photographs collected through PPM to include only those containing location information and taken within the Ishinomaki district. The survey was conducted with the approval of the Dean of the Faculty of Business Administration at Ishinomaki Senshu University. Based on the visual parameters and subject categories outlined in Table 1, Table 2, a subset of the coded results is presented in Fig. 5. These labeled data served as the basis for subsequent analyses.

#### 3.1.1 MCA results and axis interpretation

**Fig. 6:**
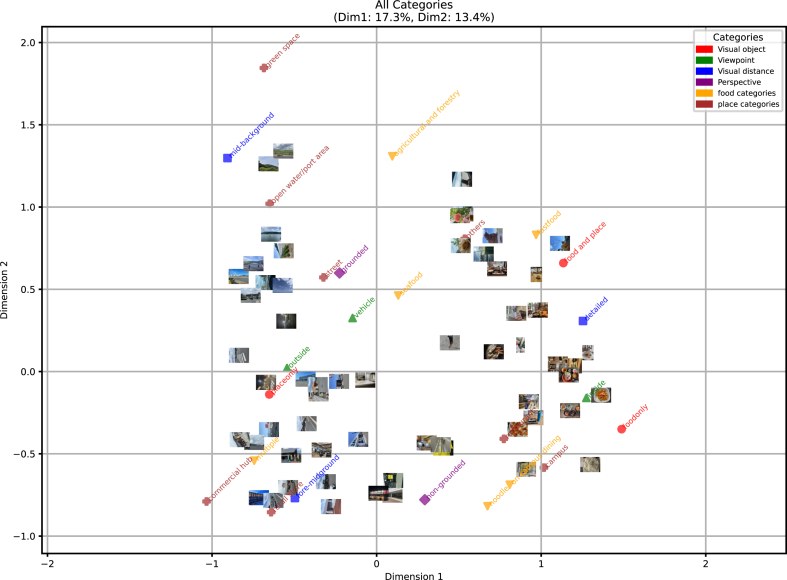
Results of MCA based on photographic components, including visual parameters (object, viewpoint, distance, and perspective), food categories, and place categories.

MCA was conducted using six items and 27 categories: four visual parameters across 11 categories, six food-related categories, and eight place-related categories. Fig. 6 presents the scatter plot illustrating the spatial configuration of category centroids on the two-dimensional plane, based on the two axes with the highest contribution rates. The list of category scores in this two-dimensional space is presented in Table 3.

**Table 3:** List of category scores on the first and second axes.

category	Dim 1	Dim 2
Visual object_foodonly	1.49	−0.35
Viewpoint_inside	1.27	−0.16
Visual distance_detailed	1.25	0.31
Visual object_food and place	1.13	0.66
place categories_campus	1.02	−0.58
food categories_fastfood	0.97	0.83
food categories_on-campus dining	0.81	−0.69
place categories_restaurant	0.77	−0.41
food categories_noodle	0.67	−0.82
place categories_others	0.54	0.81
Perspective_non-grounded	0.29	−0.78
food categories_seafood	0.13	0.46
food categories_agricultural and forestry	0.10	1.31
Viewpoint_vehicle	−0.15	0.33
Perspective_grounded	−0.23	0.60
place categories_street	−0.32	0.57
Visual distance_fore-midground	−0.50	−0.77
Viewpoint_outside	−0.54	0.02
place categories_retail store	−0.64	−0.85
place categories_open water/port area	−0.65	1.02
Visual object_placeonly	−0.65	−0.14
place categories_green space	−0.68	1.85
food categories_multiple	−0.74	−0.54
Visual distance_mid-background	−0.91	1.30
place categories_commercial hub	−1.03	−0.79

Regarding the first axis, the visual parameters contributing to directional interpretation included: the level of detail in viewing distance, the distinction between interior and exterior viewpoints, and whether the focal subject includes food. Among these, the category score differences with the largest absolute values were: “detailed”–“mid-background” (2.16), “inside”–“outside” (1.81), and “food only”–“place only” (2.14). Since photos whose main subject was food tended to be more detailed and often taken indoors, it can be inferred that the presence or absence of food is the key determinant along the first axis. For the food and place categories along this axis, only the “commercial hub” category had an absolute category score greater than 1.00. Examining the position in the scatter plot evidently revealed that photographs in the “commercial hub” category were typically taken without food, focusing only on place.

Regarding the second axis, in terms of visual parameters, the diversity of focal subjects and the absence of clear directionality in viewpoint were observed. However, for viewing distance, a directional tendency from close-up to distant views was evident, as was the presence or absence of perspective. The most notable absolute differences in category scores were “food and place”–“food only” (1.01), “mid-background”–“fore-mid-ground” (2.07), and “grounded”–“non-grounded” (1.38), suggesting that spatial depth in exterior settings plays a significant role. However, the “detailed” category was plotted near the center, indicating that viewing distance alone does not fully explain the second axis. The “green space” category exhibited an especially distinctive score of 1.85, and its strong association with “open water/port” areas and the “agricultural and forestry” food category, as well as with the “fore-midground” viewing distance, reinforces this trend. Notably, the perspective parameter appeared exclusively along the second axis. Therefore, it is interpreted that this axis reflects whether the photograph captures a broader landscape.

Based on these findings, the first axis primarily indicates whether the focal subject includes food, while the second axis reflects whether the photograph is perceived as depicting a landscape.

#### 3.1.2 Visual parameters and food categories

**Fig. 7:**
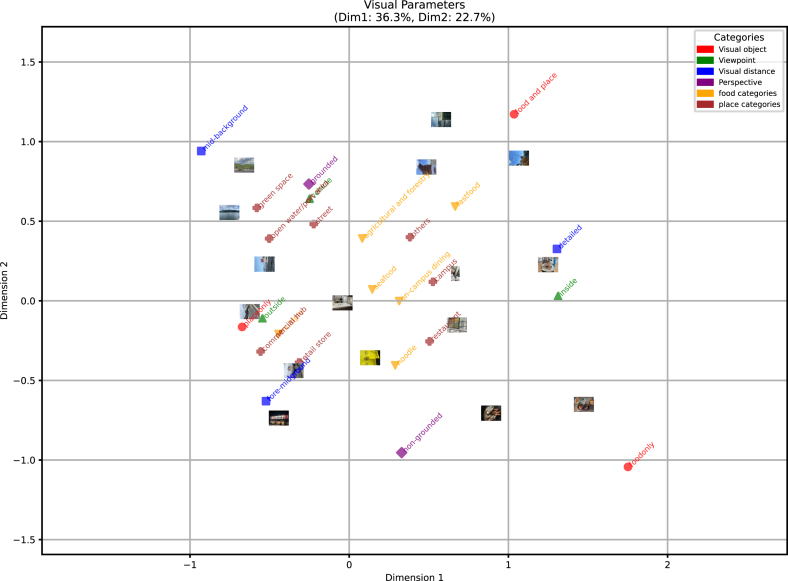
Results of MCA based on visual parameters extracted from photographs. Only visual parameters were used as active variables, while food and place categories were included as supplementary variables.

Subsequently, the visual parameters–food categories relationship was examined. To investigate this relationship, MCA was conducted using only the visual parameters, with food and place categories included as supplementary variables (see results in Fig. 7). When limited to visual parameters, the photographs were broadly categorized into 16 types. Furthermore, the relationship between the first and second axes identified earlier was preserved but had become more clearly defined.

Photographs depicting seafood products—generally recognized as emblematic of Ishinomaki—as well as university cafeteria meals, were positioned near the center of the plot. This suggests that these items are photographed in various visual contexts. Alternatively, fast food tends to be photographed in a way that captures both the food and its surrounding place simultaneously. Contrastingly, noodle dishes are more likely to be photographed with a singular focus on the food item itself, without consideration of spatial context. Additionally, although the “multiple” category comprises various items, many of these are associated with retail stores or multi-purpose facilities, and these spaces themselves are often perceived and extracted as foodscapes.

#### 3.1.3 Visual parameters and place categories

Focusing on locations in Fig. 7 restaurants are places that tend to be photographed regardless of their specific form. As mentioned earlier, retail stores and multi-purpose facilities are more likely to be photographed in the foreground and with an emphasis solely on the place itself.

#### 3.1.4 Characterizing based on hierarchical clustering

**Fig. 8:**
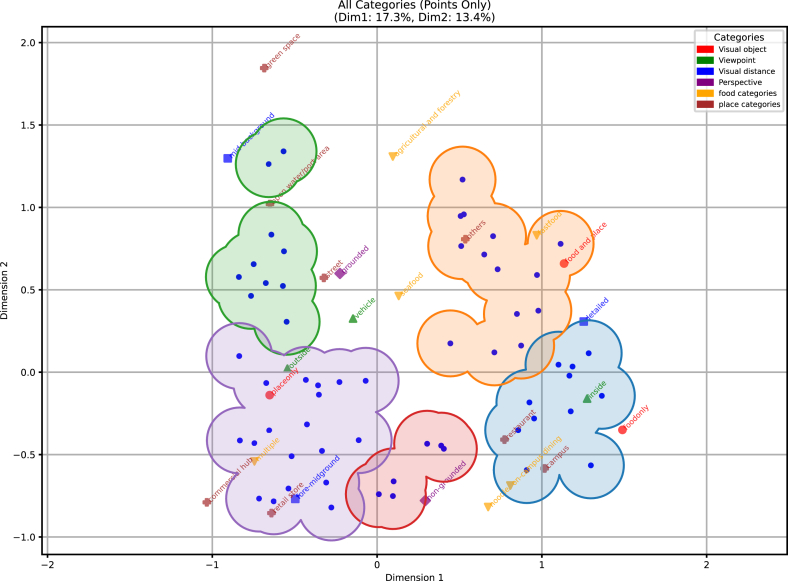
Hierarchical clustering results based on MCA (t = 2.5).

**Fig. 9:**
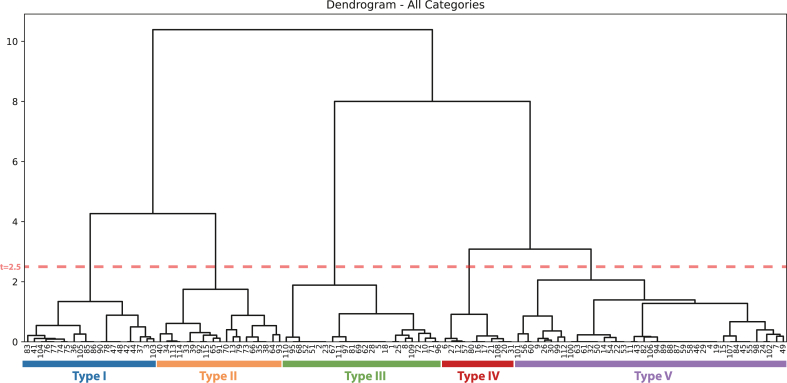
Hierarchical clustering dendrogram.

**Fig. 10:**
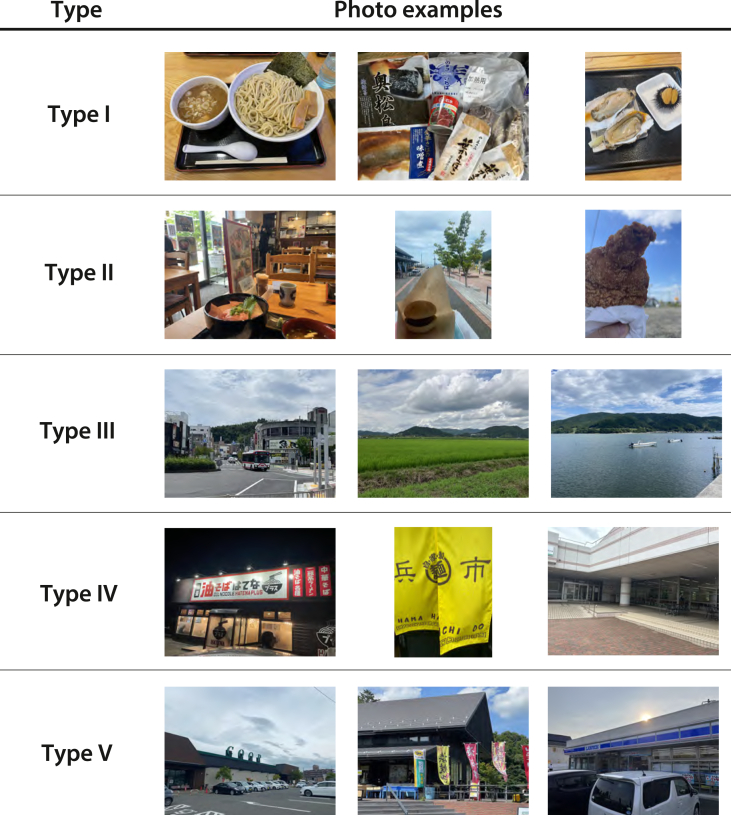
Examples of photographs for each type.

Based on the overall MCA (Fig. 6), hierarchical clustering was performed using Ward's method. The resulting clusters are plotted in the scatterplot in Fig. 8, and the dendrogram is presented in Fig. 9. To ensure enough clusters while maintaining a high degree of independence, the classification was conducted at the threshold distance of *t* = 2.5, yielding five clusters. These clusters are treated as distinct types of visual representation. Examples of photographs corresponding to each type are shown in Fig. 10.

Subsequently, the number of photographs categorized by visual target (food/place) is presented for each cluster type (Table 4). Distinctive category tendencies were observed within each type. Type I included many photographs in restaurants, with food categories being widely dispersed. This reflects the fact that photographs in Type I predominantly depicted food items alone. Type II was characterized by a predominance of fast-food photographs. In Type III, food and place categories largely corresponded, frequently representing recognizable landscapes such as the sea or agricultural areas. Type IV was primarily associated with noodle shops and university cafeterias, while Type V mainly comprised retail stores and multi-purpose complexes—spaces that handle a broad spectrum of products.

**Table 4:** Number of photographs in each type of food and place category.

	Type I	Type II	Type III	Type IV	Type V
noodle	5	0	0	8	1
seafood	4	6	9	0	4
agricultural and forestry	3	4	8	0	0
fastfood	2	9	0	0	1
on-campus dining	4	0	0	3	1
multiple	2	0	7	0	34
restaurant	13	6	0	8	8
retail store	0	1	0	0	20
street	0	3	3	0	3
commercial hub	0	0	0	0	8
campus	5	0	0	3	0
green space	0	1	8	0	0
open water/port area	1	2	11	0	1
others	1	6	2	0	1

#### 3.1.5 Geographical relationships based on five types

**Fig. 11:**
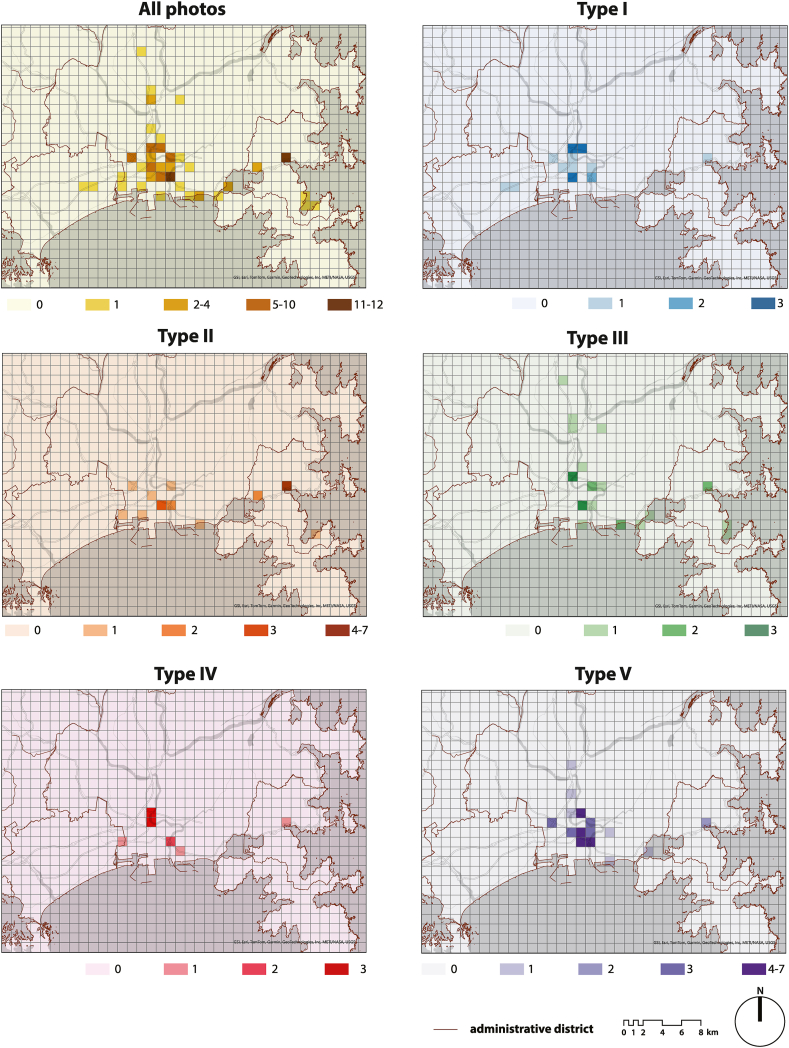
Distribution of shooting locations for each of the five types. The color intensity of each map depends on the number of shooting points contained in the grid and is graded based on an isometric distribution.

We extracted location information from the photographs, classified the photographs into the five categories mentioned above, and mapped them using ArcGIS Pro. First, Fig. 11 shows the distribution of shooting locations for each category. Since the plots overlapped due to the wide area covered, we calculated the number of photographs taken per 1 km grid and color-coded them based on equal distribution for ease of reading.

Type I was concentrated in the urban area around Ishinomaki Station. Type II was scattered throughout the urban area and coastal areas but was concentrated in specific areas such as around Onagawa Port. Type III, which included green spaces, was distributed throughout the watershed and coastal areas. Type IV was concentrated in the northern part of the city center, partly because the number of photographs was lower than for the other types. Type V was concentrated in areas even closer to the station than Types I and IV.

**Fig. 12:**
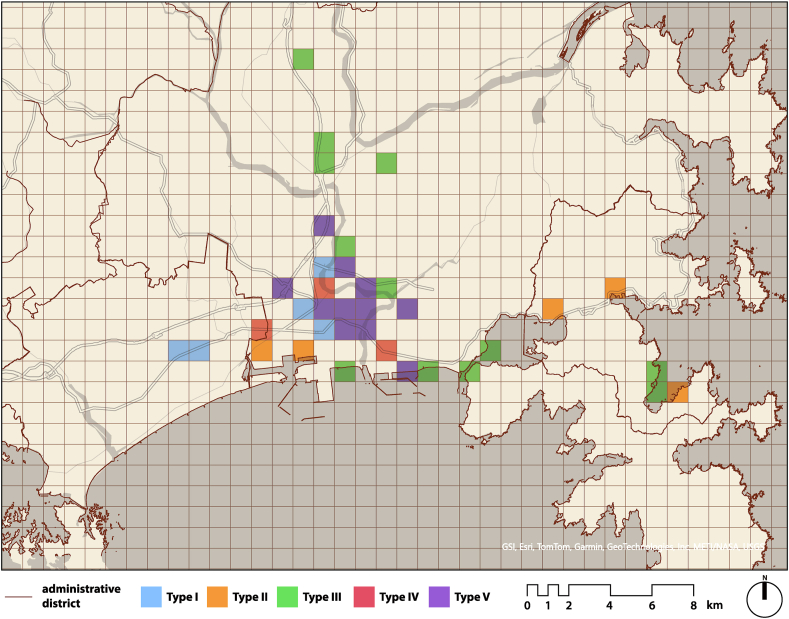
Most frequent type per 1 km grid.

Additionally, Fig. 12 shows the color coding of the most frequently detected clusters within the 1 km grid. The central area mainly contained Type V photographs, but there were also locations where Type I and IV were predominant, depending on the area. Conversely, Type II and III were found in coastal areas, and Type III had been observed inland.

### 3.2 Comparison of visual content analysis results and preliminary survey results

Table 5, Table 6 present a comparison between the PPM results and the preliminary questionnaire findings for the food and place categories, respectively.

**Table 5:** Percentage of food categories selected as characteristic of the Ishinomaki district.

	noodle	seafood	Agricultural and forestry	fastfood	on-campus dining	multiple
Questionnaire (n = 117)	10.3 %	82.1 %	6.8 %	0.0 %	0.0 %	0.9 %
Photos (n = 115)	10.8 %	17.7 %	11.5 %	9.2 %	6.2 %	33.1 %

**Table 6:** Percentage of place categories selected as characteristic of the Ishinomaki district.

	restaurant	retail store	street	commercial hub	campus	green space	open water/port area	others
Questionnaire (n = 108)	0.9 %	0.9 %	6.5 %	38.0 %	1.9 %	13.0 %	18.5 %	20.4 %
Photos (n = 115)	28.2 %	16.9 %	7.3 %	6.5 %	6.5 %	7.3 %	12.1 %	8.1 %

Regarding food, while over 80 % of respondents identified marine products as characteristic local specialties of the Ishinomaki district in the questionnaire, photographs related to seafood and the marine landscape (e.g., seafood dishes, the sea, ports) made up only 17.7 % of the total photographs. Notably, the photographic method led to a greater diversity in the food categories represented.

For place categories, whereas the questionnaire responses were dominated by commercial hubs and others, most photo-based responses depicted restaurants and retail shops. Notably, the “commercial hub” mentioned in the questionnaires included food-related venues such as Ishinomaki Genki Ichiba, alongside institutions like museums. The “others” category in the questionnaire often referred to recreational spaces or historical buildings. The “green space” was referenced in both the textual and visual data; however, while green spaces mentioned in the text—particularly as regional landmarks—primarily referred to mountains and islands, those depicted in the photographs were mostly agricultural fields.

Overall, the visual representations captured through photographs exhibited less categorical concentration, suggesting a broader and more varied depiction compared to the verbal responses.

## 4 Discussion

### 4.1 Food and landscape in the ishinomaki district

In Ishinomaki district, food and landscape exhibit distinct characteristics across urban, natural, marine, rural, and forest settings. Visual analysis identified five foodscape types (I–V), collectively revealing the relationship between food and place. Type I photographs, depicting food in isolation, reflect culturally familiar meals tied to daily life and memory rather than local identity. Types II–V, combining food with spatial elements or landscapes, connect culinary culture to both consumption and production, highlighting the multi-layered nature of local foodscapes.

Spatial patterns show that food-place photographs cluster at iconic sites such as Onagawa Seapal- Pier, whereas landscape-focused photographs are distributed along rivers or other accessible locations. Combining food and place often reflects tourist-oriented locations, whereas landscape-focused photographs emerge from habitual use, indicating that environment in daily settings shape perceptions. Comparisons with textual responses reveal divergences: marine products dominate texts, whereas photographs capture everyday meals, fast food, and campus contexts, with green spaces emerging visually despite rare textual mention. Visual methods thus capture multilayered, culturally grounded perceptions.

Overall, Ishinomaki's foodscapes intertwine cultural memory, daily practices, and environmental context. Integrating visual and textual approaches provides a nuanced understanding of locally grounded foodscapes.

### 4.2 Contributions to locally grounded foodscape research

Following Vonthron et al. (2020), studies focusing on individual perceptions, including this one, fall under behavioral approaches. These approaches leverage foodscape concepts and spatial analysis (Mikkelsen, 2011; Earl, 2018), often supported by GPS and GIS (Mikkelsen, 2011). However, spatial analyses have mainly examined health outcomes via food access, while behavioral approaches focus on practices in institutional facilities, households, or retail stores, limiting their spatial scope. Macro-scale studies, in contrast, risk deterministic interpretations of dietary behavior (Sobal and Wansink, 2007). This study bridges micro- and macro-scales and spatial and behavioral dimensions by analyzing perceptions across multiple municipalities.

Fast food, frequently addressed in spatial studies due to health implications (Lebel et al., 2012; Eckert and Vojnovic, 2017), is treated here as a low regional specificity food type. Visual analysis shows it is frequently associated with spatial elements, suggesting Type II photographs reflect a simple food-place association and latent perceptual connections. Visual methods thus complement access-based spatial analyses, enriching understanding of community foodscapes (Chen and Clark, 2016).

Socially grounded approaches typically emphasize descriptive analysis, cultural practices, or policies (Holloway and Kneafsey, 2000; Miewald and McCann, 2014). Our study demonstrated that combining visual and textual data reveals latent linkages between food and place not easily expressed linguistically, extending visualization methods from cognitive mapping (Earl, 2018; Tolman, 1948). In Ishinomaki district, texts highlight local specialties, while photographs depict daily meals, fast food, and campus contexts, demonstrating that lived experience and daily accessibility shape foodscapes beyond “specialty products.”

Subjective and culturally embedded perceptions, such as attachment to school cafeterias or local shops, are as central as iconic landscapes. This perception-oriented, multimodal perspective extends foodscape research and provides new insights into locally grounded foodscapes.

### 4.3 Perspectives on food in landscape planning and tourism

This study adapts methods from tourism and landscape research to foodscape scholarship, using visual analysis to explore links between tourism, landscape planning, and locally embedded food practices.

The visual and landscape perspective has gained salience in foodscape research (Sobal and Wansink, 2007; Mikkelsen, 2011). Roe et al. (2016) applied Landscape Character Assessment to Newcastle upon Tyne, and integrated visual and landscape perspectives with public approaches, which are considered equally important as expert evaluation in landscape studies (Zube et al., 1982b; Arthur et al., 1977; Daniel et al., 1983), through consultation and dialogue. In contrast, our study employs data directly perceived by residents, positioning resident participation as a public approach. By doing so, we obtained datasets that are more appropriate for statistical analysis. While qualitative interpretations obtainable only through dialogue or deliberation and the risks associated with numerical weighting have been noted (Landscape Institute and Institute of Environmental Management and Assessment, 2013), this study is grounded in expert qualitative interpretations, which are rendered into data to allow systematic summarization. In this way, the method offers a distinct advantage in its ability to visualize the qualitative meanings of foodscapes embedded in local contexts.

In tourism research, communities and residents who experience environmental changes firsthand are crucial stakeholders (Li et al., 2019). Food tourism can foster community development (Park and Widyanta, 2022). Digitalization of tourism information and social media influence urban foodscapes (Goodman and Jaworska, 2020; Chen et al., 2023), giving rise to new area of study, “digital foodscapes” (Joassart-Marcelli, 2025).

While digital platforms have expanded opportunities for tourism promotion, they tend to privilege prominent sites, often overlooking local foodscapes of everyday significance. In Ishinomaki, for instance, supermarkets and fast food outlets, which are rarely featured in tourist guides, emerged as meaningful elements of residents’ daily lives, revealing a gap between tourist representations and lived local perceptions. Incorporating such landscapes in daily settings and food outlets, together with established attractions such as Onagawa Seapal Pier, into tourism promotion may contribute to more inclusive and sustainable forms of food tourism.

Advances in machine learning also suggest potential for future study: perceptual data from this study could serve as AI training datasets, offering new methodological contributions to regional research.

## 5 Conclusion

This study examined the visual representations and perceptions of the “foodscape” in the Ishinomaki district by analyzing visual content and comparatively investigating linguistic and non-linguistic representations. Resultantly, five typologies based on visual scale were identified, comparative analysis of which revealed that the foodscape is represented not merely by the presence of food, but is intricately linked with diverse elements such as place, memory, everyday life, and regional identity. Particularly, focusing on the food–place relationship, there was observed a tendency to associate food with natural landscapes and symbolic facilities, suggesting that the visual foodscape reflects personal experiences rooted in daily life.

Furthermore, a certain divergence was found between linguistic and visual representations. While foods identified as “specialties” in textual responses relied on fixed regional images such as marine products, the photographs encompassed a broader variety of more commonplace meals, confirming that visual representations more strongly reflect lived experience. This supports the notion that representations are closely intertwined with subjective factors such as cognition, memory, and experience.

Moreover, elements such as accessibility and familiarity influenced visual representations through photography. Additionally, the question formats differed in nature: linguistic responses were prone to social value judgments such as “recommendation” and “pride,” whereas visual responses involved selective behavior inherent in the act of photographing, resulting in differing data characteristics.

These findings illustrate that understanding regional food and landscape requires attention to visual and experiential dimensions that cannot be fully captured by linguistic data alone. An integrated analysis of both enables a more nuanced and multidimensional understanding of the region. This study demonstrated that analyzing visual representations is effective for comprehending the multilayered culture and practices rooted in local contexts.

By systematically incorporating non-verbal representations of foodscapes, this study provides an empirical basis for further investigation into how everyday spatial contexts influence food-related perceptions and associations. This analytical perspective offers a framework for developing more context-sensitive, resident-informed approaches in the fields of food tourism, regional planning, and cultural landscape research.

As an illustration, the World Food Travel Association defines food tourism as “the act of traveling for a taste of place in order to get a sense of place (World Food Travel Association, 2015).” From this perspective, the findings of this study also have important implications for food tourism: by visualizing the connections between taste and place that are embedded in local perceptions—often distinct from generalized notions of regional specialties—this research offers a way to uncover more authentic, locally grounded foodscape experiences. As such, it contributes to a more essential understanding of food tourism rooted in everyday cultural practices.

We place our foodscape research within broader the context of visual analytical methods in landscape studies as theoretical contribution. Locally grounded foodscape research tended to focus on specificities of individual cases which are more universalized here. The applied methodology demonstrates the potential for analyzing locally grounded foodscapes based on both linguistic and non-linguistic perspectives. The results can integrate perceptions of local residents into tourism planning, thereby offering a framework for tourism management reflecting perfectives of both visitors and local communities. These theoretical insights extend multimodal research on food, particularly in neuroscience (e.g. (Rolls and Baylis, 1994; Verhagen and Engelen, 2006),), by presenting applied outcomes derived directly from residents’ perspectives that are adaptable to macro-scale contexts.

For more practical purposes, the integration of visual and textual analyses here provides planners, food tourism developers, and community organizations with a framework for understanding how residents experience and perceive their local food environments. The results can serve as an inventory for destination management and contribute to co-creating dynamic and food tourism with local communities.

Alternatively, this study has certain methodological limitations, which are dependence on expert judgements the limited number of data points, the lack of comparisons with visitors and local residents, and limited scope of respondents to university students. Expert-based evaluations beyond purely numerical measures are increasingly adopted in the field of landscape assessment (Landscape Institute and Institute of Environmental Management and Assessment, 2013) and this paper aimed to conduct such evaluations by combining both visual and textual analyses. In order to further generalization beyond the study area, the methodology requires expanding both the quantity of evaluative data and the sample size. Additionally, comparative analysis with visitors' visual perceptions could help identify destinations that are uniquely salient to local residents. Moreover, as the participants were limited to university students, it is difficult to claim that their views fully represent those of the Ishinomaki district's residents, and the presence of photographs of fast food and campus cafeterias may reflect an attribute bias. By collecting photographic data from more diverse range of age groups and social actors, future research can obtain more generalizable results. Furthermore, methodology of computational visual content analysis can be applied based on computer vision to create model for foodscape image classification in future study. Such a model can facilitate intergenerational and regional comparisons of the visual dimensions of foodscapes when combined with large-scale datasets.

## Author contributions

Masaki Uchida: Writing – original draft, Conceptualization, Visualization, Validation, Methodology, Formal analysis, Data curation. Rei Sugawara: Investigation, Resources, Data curation. Ryo Kohsaka: Writing – review & editing, Supervision, Project administration, Funding acquisition.

## Funding sources

This work is supported and funded by JSPS KAKENHI [grant numbers JP22H03852; JP23H01584; JP23H03605]; JST [grant number JPMJPF2110]; Environment Research and Technology Development Fund of the Environmental Restoration and Conservation Agency provided by Ministry of the Environment of Japan [grant number JPMEERF 20241M03]. The funding sources had no involvement in the design, execution, or interpretation of the research.

## Declaration of competing interest

The authors declare the following financial interests/personal relationships which may be considered as potential competing interests: Masaki Uchida reports financial support was provided by 10.13039/501100002241Japan Science and Technology Agency. Ryo Kohsaka reports financial support was provided by 10.13039/501100002241Japan Science and Technology Agency. Masaki Uchida reports financial support was provided by 10.13039/100014423Environmental Restoration and Conservation Agency. Ryo Kohsaka reports financial support was provided by 10.13039/100014423Environmental Restoration and Conservation Agency. Ryo Kohsaka reports financial support was provided by 10.13039/501100001691Japan Society for the Promotion of Science. If there are other authors, they declare that they have no known competing financial interests or personal relationships that could have appeared to influence the work reported in this paper.

## Data Availability

Data will be made available on request.
